# Supplementary figures and images for: Inhibition of FLT1 ameliorates muscular dystrophy phenotype by increased vasculature in a mouse model of Duchenne muscular dystrophy
Source: PLoS Genet. 2019 Dec 26;15(12):e1008468. doi: 10.1371/journal.pgen.1008468 (PMC6932757; doi:10.1371/journal.pgen.1008468)

S1 Fig

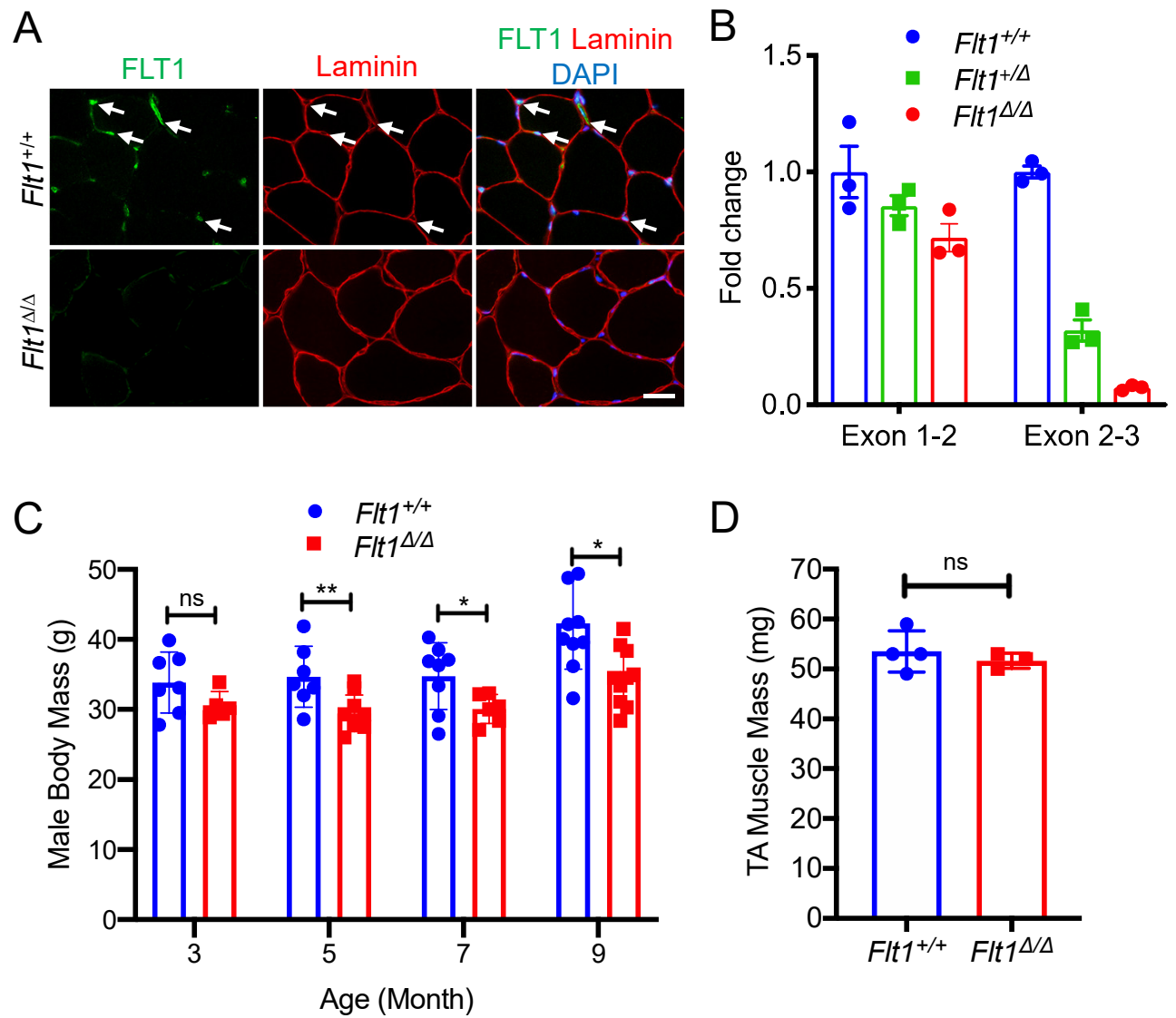

Supplement: S1 Fig — A. Immunostaining for FLT1 (green, arrows) and Laminin (red) shows effective deletion of FLT1 in TA muscle of Flt1Δ/Δ mice following tamoxifen (TMX) injection. DAPI staining (blue) is for all nuclei. Scale bars indicate 20 μm. B. RT-qPCR shows deletion of the Flt1 exon 3 in the TA muscle of Flt1Δ/Δ mice while exons 1 and 2 are retained. C. Flt1Δ/Δ male mice show no difference in the body mass during the time course D. Flt1Δ/Δ male mice show no difference in TA muscle mass compared to Flt1+/+ male mice. (PDF) [file pgen.1008468.s001.pdf]

S3 Fig

A

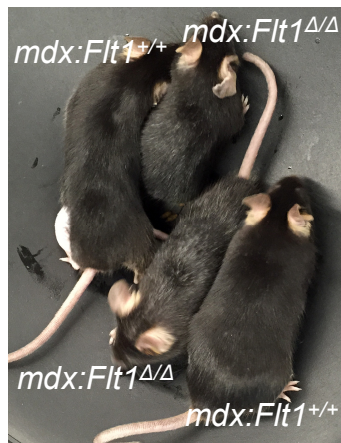

B

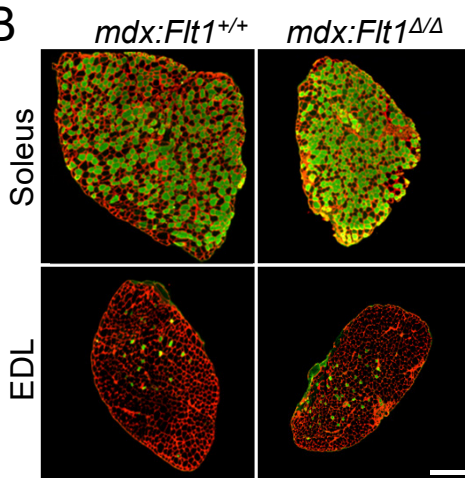

C

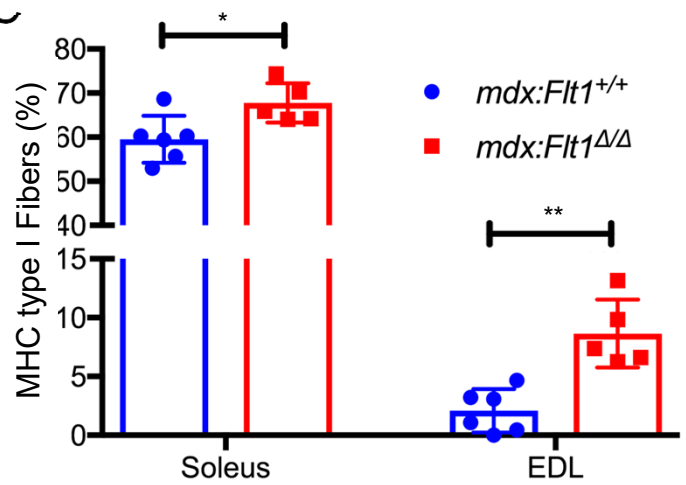

D

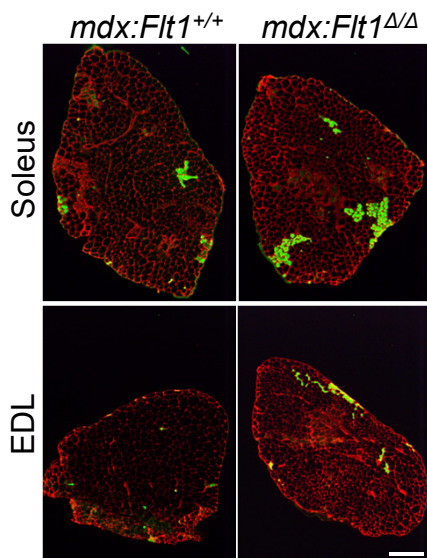

E

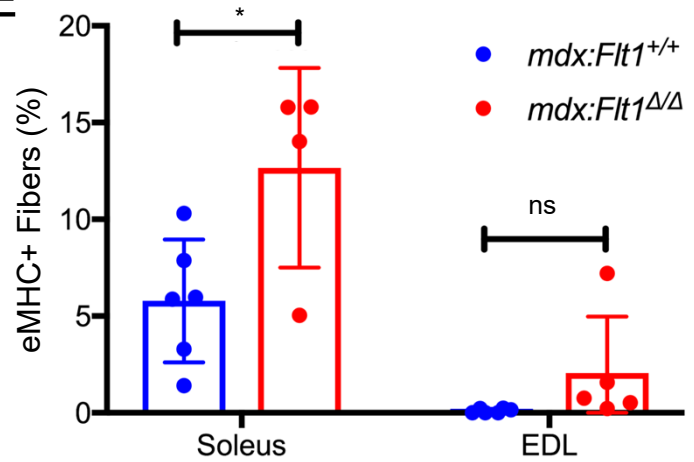

Supplement: S3 Fig — A. mdx:Flt1Δ/Δ mice show signs of premature aging such as white hair. B. Representative image of type I slow MHC (green) and Laminin (red) in EDL and soleus muscle in mdx:Flt1+/+ and mdx:Flt1Δ/Δ mice. Scale bars indicate 200 μm. C. Type I slow MHC+ fibers are increased in the mdx:Flt1Δ/Δ mice in both EDL and Soleus muscle. D. Representative image of eMHC (green) and Laminin (red) in EDL and soleus muscle in mdx:Flt1+/+ and mdx:Flt1Δ/Δ mice. Scale bars indicate 200 μm. D. eMHC staining shows decreased fiber stability in muscle fibers in the soleus but not the EDL muscle in the mdx:Flt1Δ/Δ mice. (PDF) [file pgen.1008468.s003.pdf]

S4 Fig

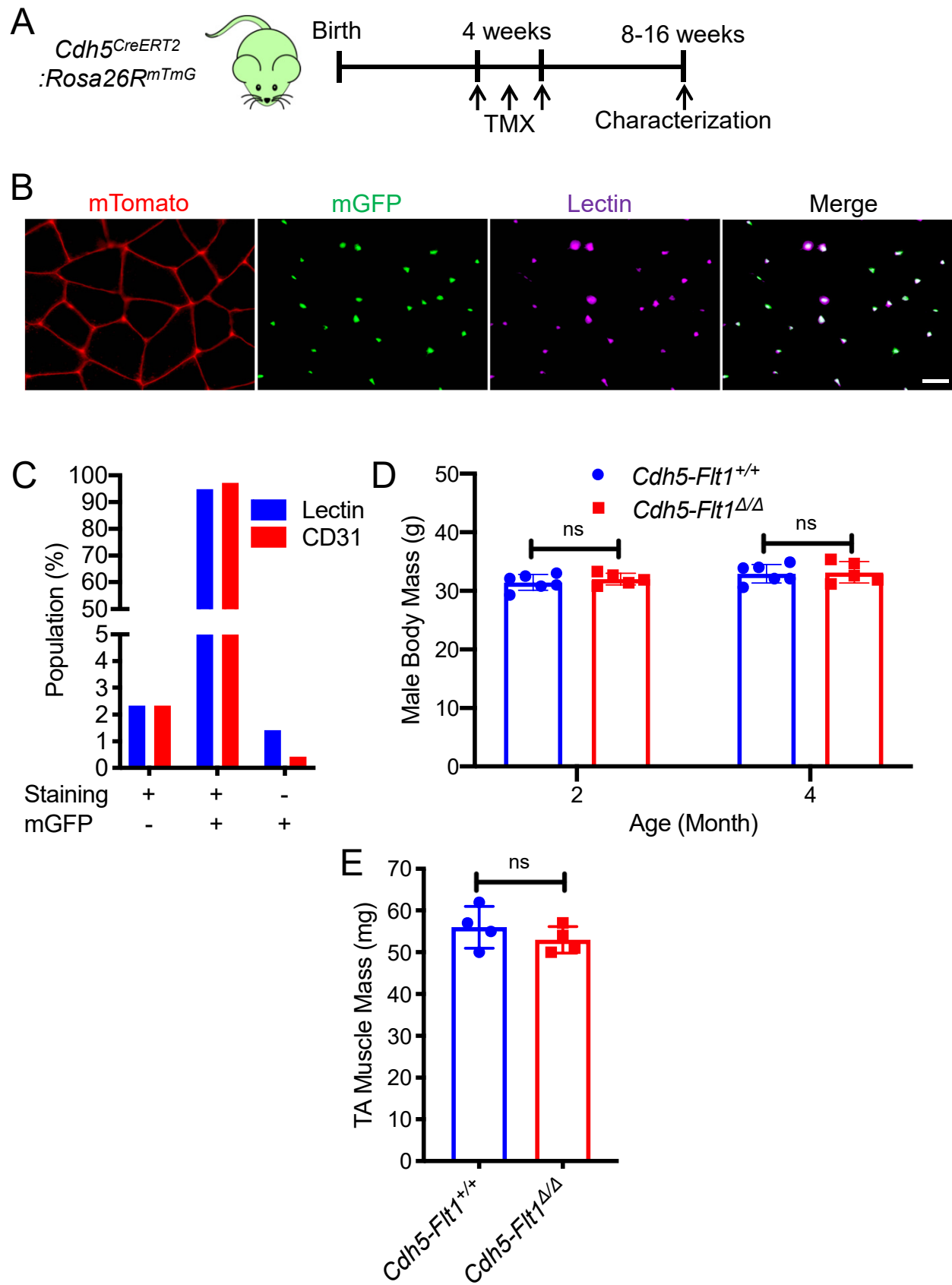

Supplement: S4 Fig — A. Experimental scheme for assessing angiogenic response from conditional Flt1 deletion. B. Cdh5CreERT2:Rosa26RmTmG mice reveal that Cdh5CreERT2 efficiently induced mGFP expression in the capillaries (green) labeled with lectin (purple), but not in other cell types including muscle fibers (red). Scale bars indicate 20 μm. C. Cdh5CreERT2:Rosa26RmTmG mice show efficient mGFP labeling of lectin+ and CD31+ endothelial cells. Histogram indicates that more than 90% of the cells are CD31+mGFP+ or lectin+mGFP+. D.E. Body mass and TA muscle mass are unchanged in Cdh5-Flt1+/+ and Cdh5-Flt1Δ/Δ mice. (PDF) [file pgen.1008468.s004.pdf]

S5 Fig

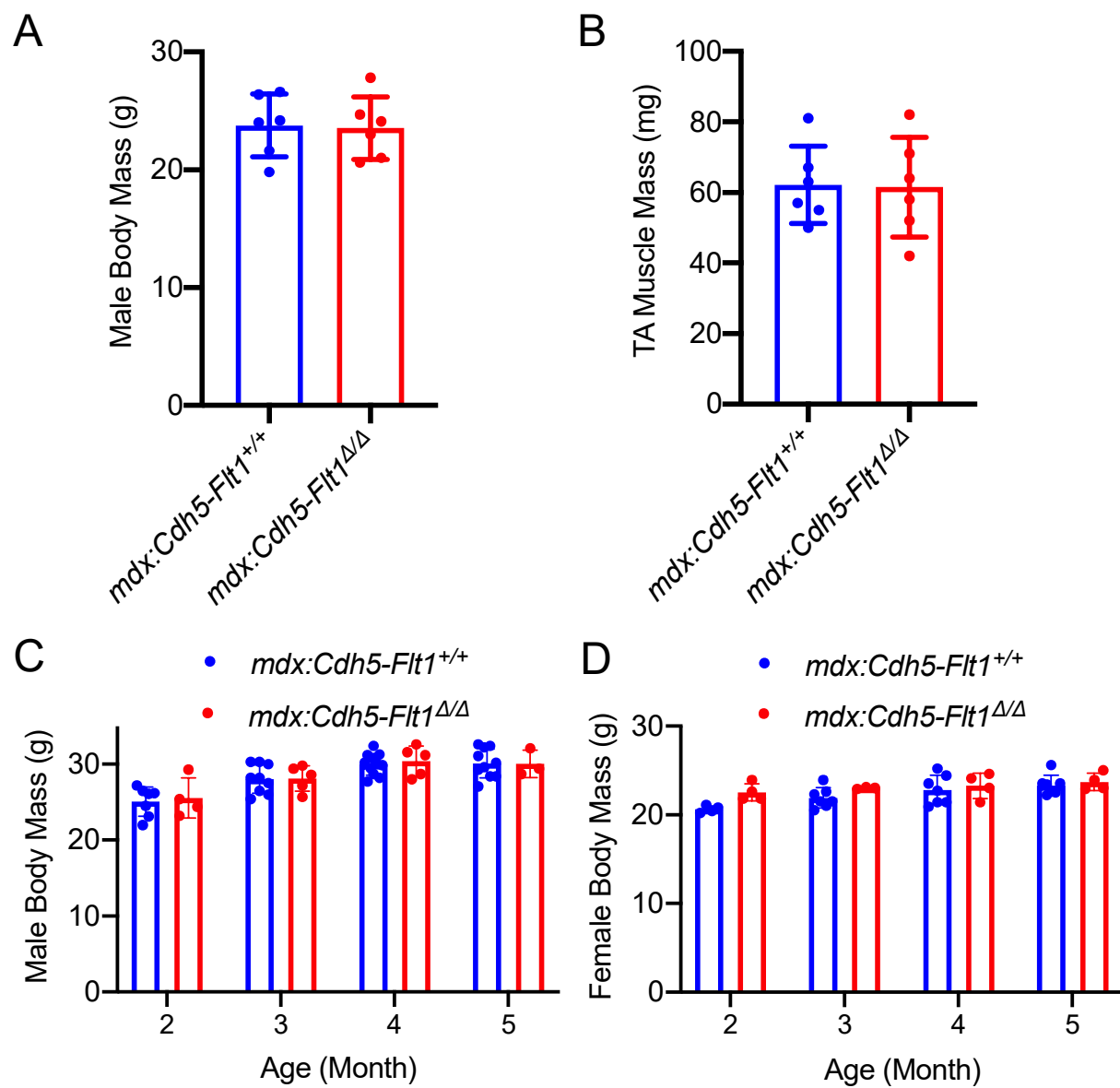

Supplement: S5 Fig — A. B. Body mass and TA muscle mass are unchanged in mdx:Cdh5-Flt1+/+and mdx:Cdh5-Flt1Δ/Δ mice. C. D. Body mass is unchanged in male or female mdx:Cdh5-Flt1+/+and mdx:Cdh5-Flt1Δ/Δ mice during the time course. (PDF) [file pgen.1008468.s005.pdf]

S6 Fig

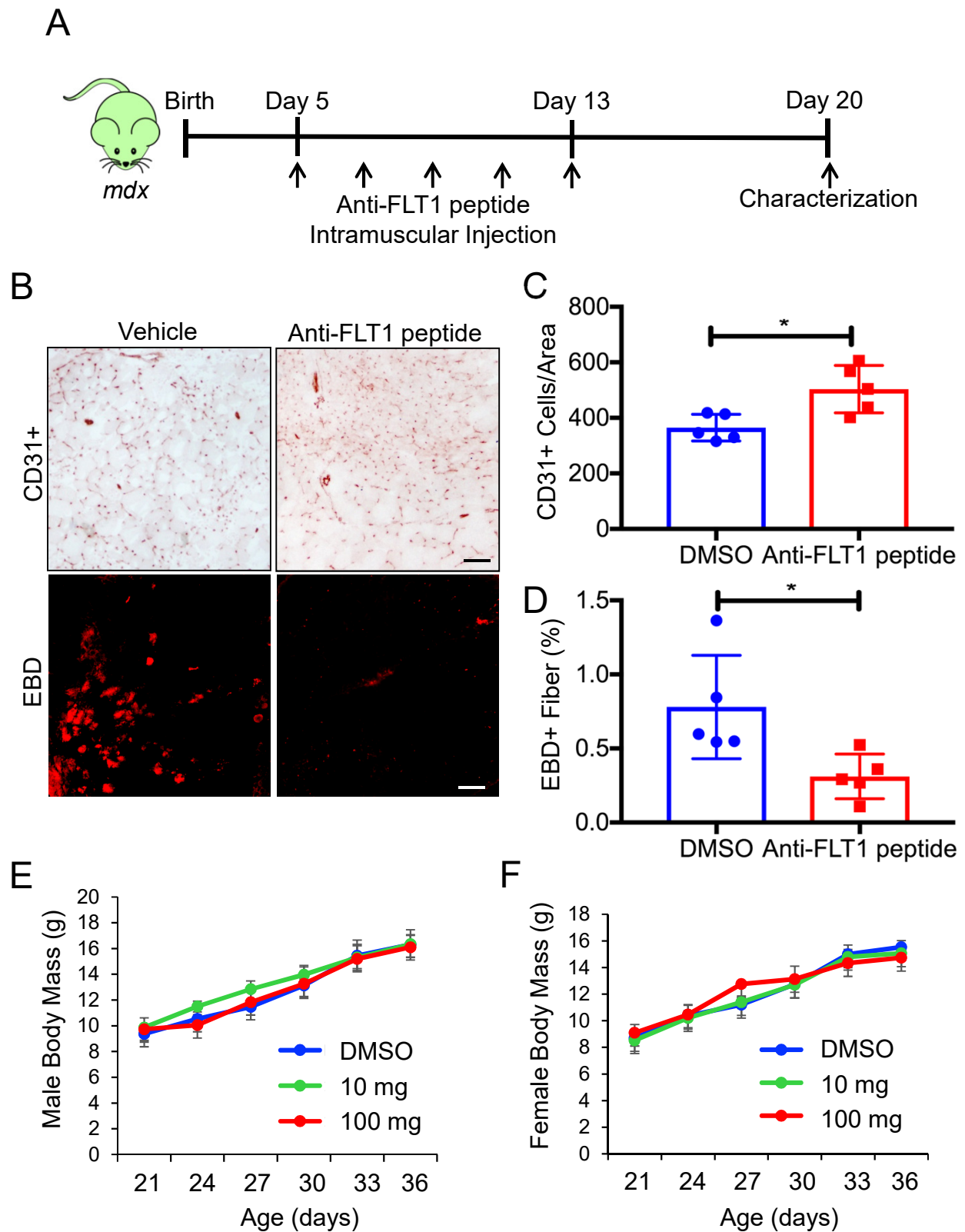

Supplement: S6 Fig — A. Experimental scheme for proof of principle study of mdx mice with intramuscular injection of anti-FLT1 peptide. B. Representative images of CD31 (top) and EBD staining (bottom) of TA muscle injected with anti-FLT1 peptide. Scale bars indicate 50 μm. C. Neonatal intramuscular injection of anti-FLT1 peptide increases capillary density in the TA muscle of the mdx mice. D. Neonatal intramuscular injection of anti-FLT1 peptide decreases EBD+ area in the TA muscle of the mdx mice. E. F. Systemic anti-FLT1 peptide injection does not change body mass in the male or female mdx mice at low or high dose. (PDF) [file pgen.1008468.s006.pdf]

S7 Fig

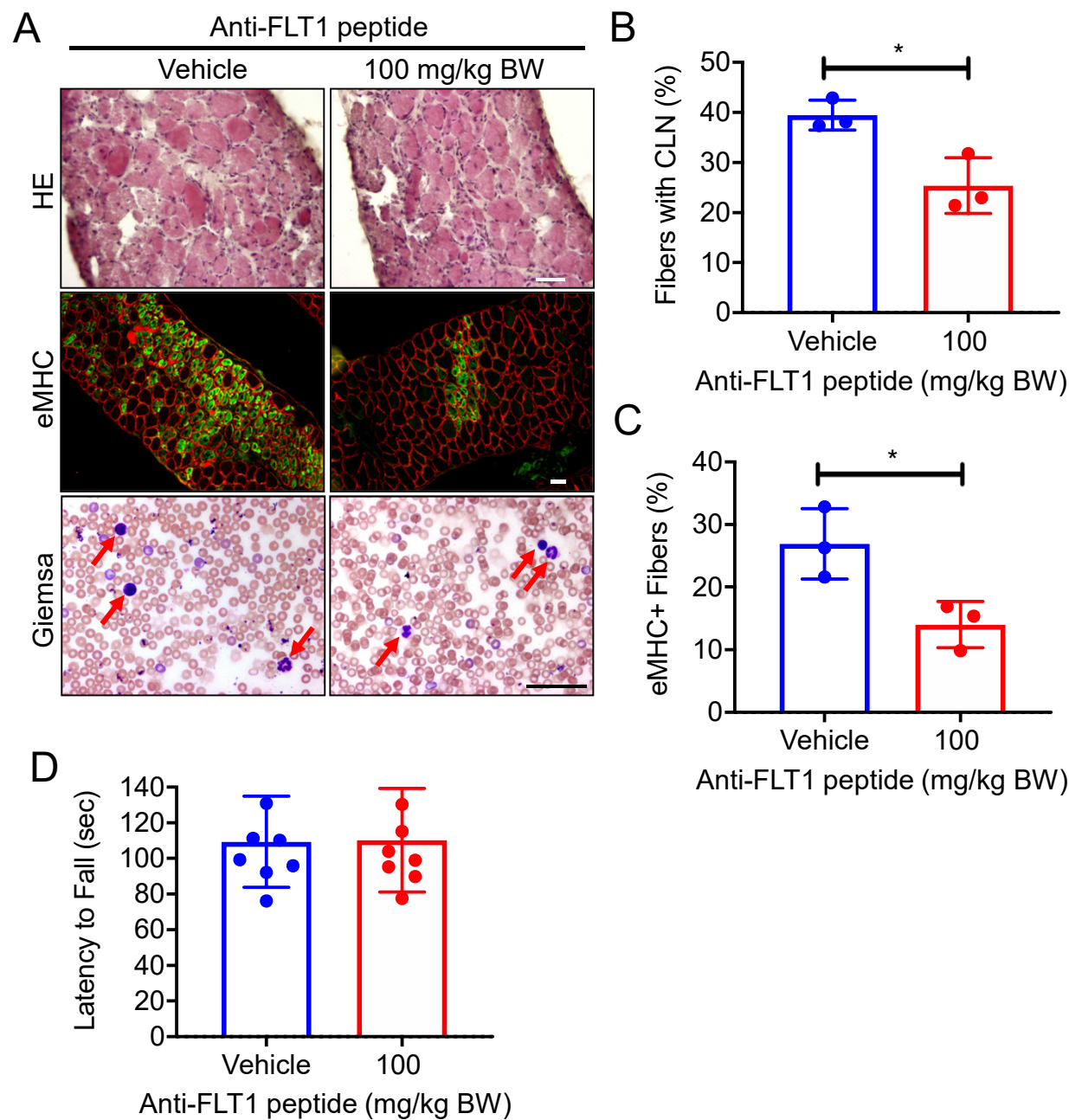

Supplement: S7 Fig — A. Representative images of HE staining, eMHC staining of diaphragm and Giemsa staining of blood smears in mdx mice treated with anti-FLT1 peptide. Allows indicate myeloid cells and lymphocytes. Scale bars indicate 100 μm. B. Diaphragm muscle fiber turnover is reduced in mdx mice treated with anti-FLT1 peptide as evaluated by centrally located nuclei (CLN). C. eMHC staining shows increased fiber stability in muscle fibers in the diaphragm of mdx mice treated with anti-FLT1 peptide. D. No difference in motor coordination or balance on the Rotarod was observed between the groups. (PDF) [file pgen.1008468.s007.pdf]

S8 Fig

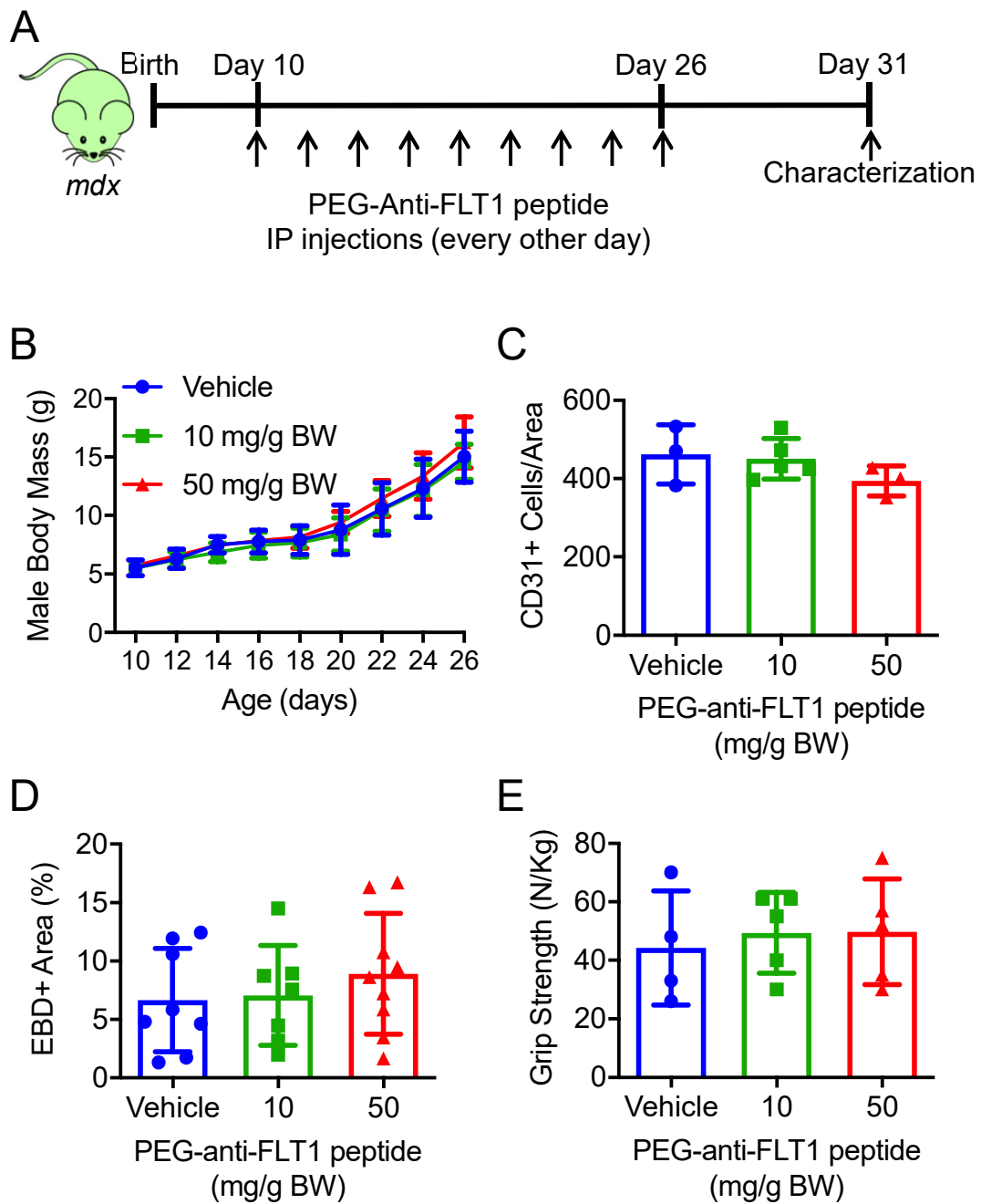

Supplement: S8 Fig — A. Experimental scheme for systemic treatment of mdx mice with IP injection of PEG-anti-FLT1 peptide. B. Systemic PEG-anti-FLT1 peptide injection does not change body mass in the male mdx mice at low or high dose. C. Systemic PEG-anti-FLT1 peptide injection does not increase capillary density in the mdx mice at low or high dose. D. Systemic PEG-anti-FLT1 peptide injection does not decrease EBD in the mdx mice at low or high dose. E. Systemic PEG-anti-FLT1 peptide injection does not improve grip strength in the mdx mice at low or high dose. (PDF) [file pgen.1008468.s008.pdf]

S9 Fig

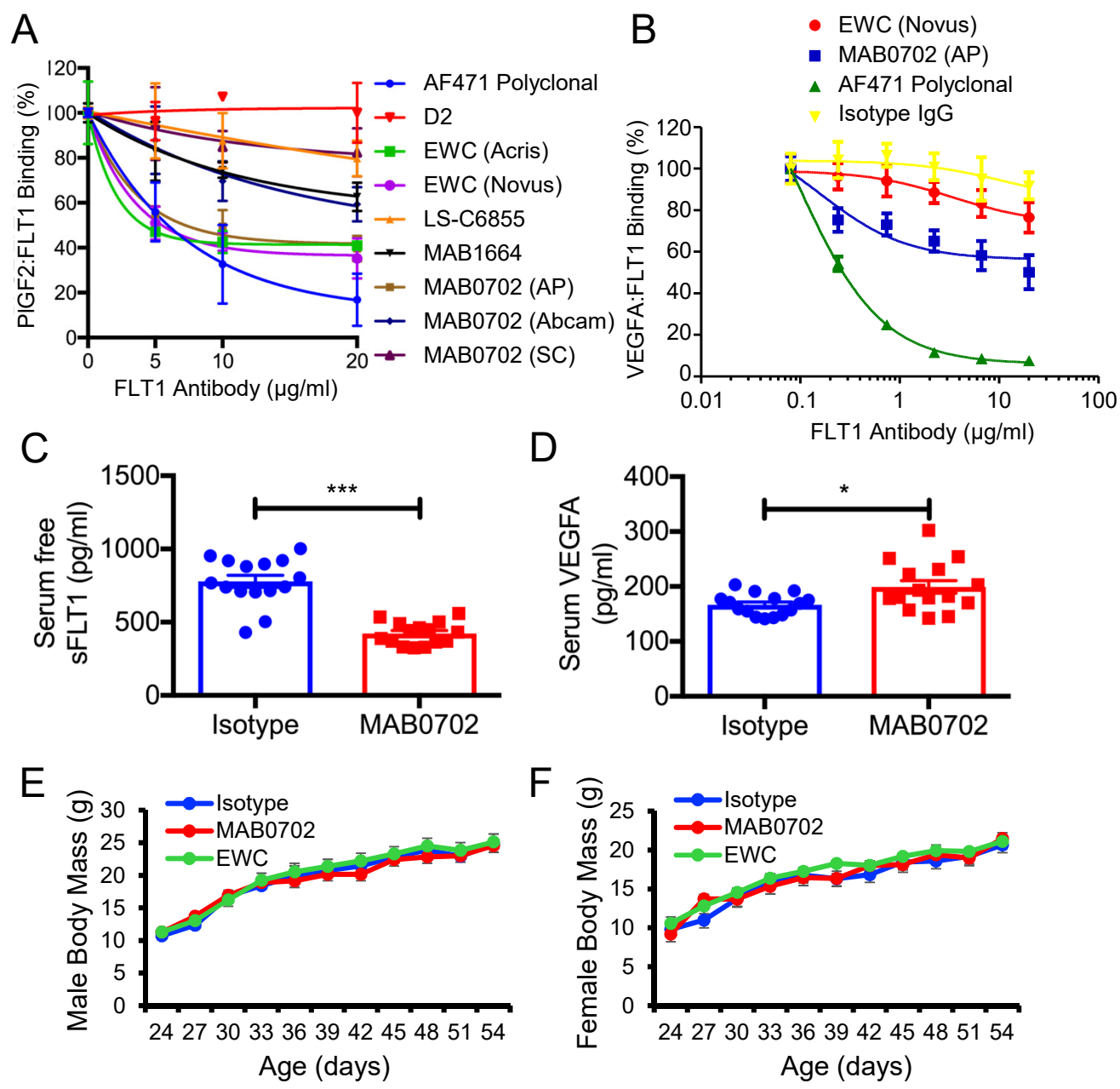

Supplement: S9 Fig — A. Commercially available MAbs for anti-FLT1 screened for blocking activity against PlGF using ELISA. AF471 polyclonal anti-FLT1 antibody was used as a positive control. AP, Angio-Proteomie; SC, Santa Cruz Biotechnology. B. Two selected MAbs screened for blocking activity against VEGFA using ELISA. AF471 polyclonal anti-FLT1 antibody was used as a positive control. AP, Angio-Proteomie. C. Serum free sFLT1 is decreased in mice injected with MAB0702 compared to isotype control. D. Serum free VEGFA is increased following MAB0702 treatment. E. F. Systemic anti-FLT1 antibody injection does not change body mass in the male or female mdx mice. (PDF) [file pgen.1008468.s009.pdf]
