# Supplementary material for: Inhibition of FLT1 ameliorates muscular dystrophy phenotype by increased vasculature in a mouse model of Duchenne muscular dystrophy
Source: PLoS Genet. 2019 Dec 26;15(12):e1008468. doi: 10.1371/journal.pgen.1008468 (PMC6932757; doi:10.1371/journal.pgen.1008468)

S2 Fig

A

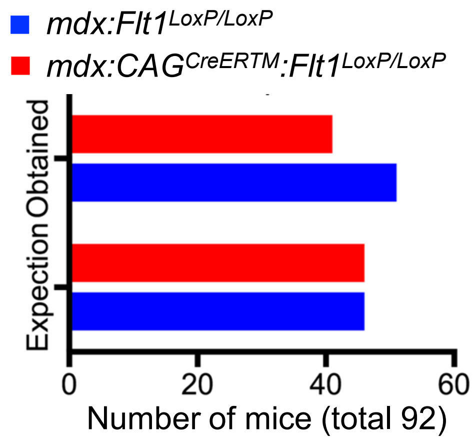

B

	Viability 5 days Post Tamoxifen Injection			
	mdx:Flt1$^{LoxP/LoxP}$		*mdx:CAG*^{CreERTM}*:Flt1*$^{LoxP/LoxP}$	
Tamoxifen Start Age	Vehicle	TMX or 4-OHT	Vehicle	TMX or 4-OHT
Postnatal day 3 (p3)	N/A	12/14 (TMX)<br>8/8 (4-OHT)	N/A	0/8 (TMX)<br>0/10 (4-OHT)
p5	N/A	7/8 (TMX)	N/A	0/7 (TMX)
p16	N/A	9/9 (TMX)	13/13	5/11 (TMX)
p21	N/A	8/8 (TMX)	N/A	10/10 (TMX)
p26	N/A	N/A	6/6	6/6 (TMX)
p31	N/A	5/5 (TMX)	6/6	7/7 (TMX)
p240	N/A	6/6 (TMX)	N/A	7/7 (TMX)

C

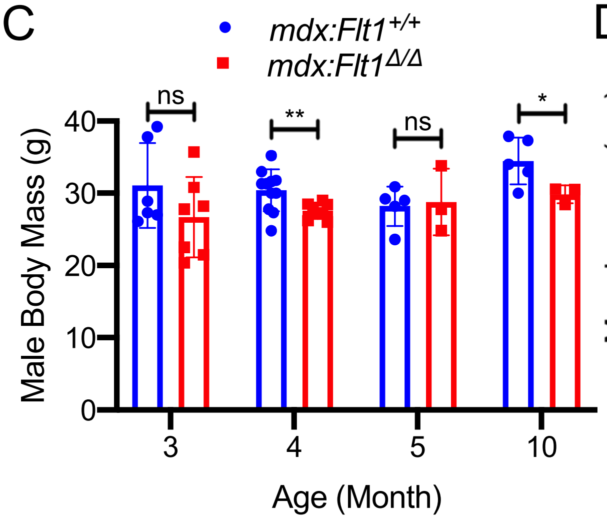

D

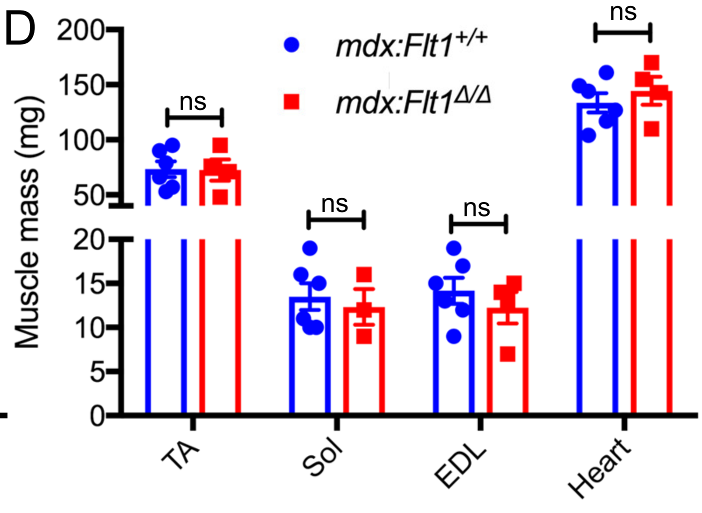

Supplement: S2 Fig — A. mdx:CAGCreERTM:Flt1LoxP/LoxP mice breed with mdx:Flt1LoxP/LoxP mice, and yield mice in expected ratios in a total of 92 mice genotyped. Chi-squared test shows no difference in expected. B. Induction of CreERTM by TMX or 4-hydroxy-tamoxifen (4-OHT) shows that Flt1 deletion in mdx mice prior to p21 results in partial or complete lethality in the mdx:Flt1Δ/Δ but not control Flt1+/+ mice. C. mdx:Flt1Δ/Δ mice show reduced male body mass but no difference in (D) muscle mass. (PDF) [file pgen.1008468.s002.pdf]
