# Supplementary material for: Inhibition of FLT1 ameliorates muscular dystrophy phenotype by increased vasculature in a mouse model of Duchenne muscular dystrophy
Source: PLoS Genet. 2019 Dec 26;15(12):e1008468. doi: 10.1371/journal.pgen.1008468 (PMC6932757; doi:10.1371/journal.pgen.1008468)
Supplement: S1 Table — (PDF) [file pgen.1008468.s010.pdf]

Table S1

Table S1. Summary of mice used for this paper

Mouse model	Treatment	Figure	Vascular change	Muscle pathology	Significant functional outcome
CAG^{CreERTM}*:Flt1*$^{LoxP/LoxP}$ (*Flt1*$^{\Delta/\Delta}$)	Tamoxifen (TMX)	1, S1	Increased capillary density	Not evaluated	Lower body mass
mdx:CAG^{CreERTM}*:Flt1*$^{LoxP/LoxP}$ (*mdx:Flt1*$^{\Delta/\Delta}$)	TMX	2, S2,S3	Increased capillary density	Worse muscle pathology	Lower body mass
Cdh5^{CreERT2}*:Flt1*$^{LoxP/LoxP}$ (*Cdh5-Flt1*$^{\Delta/\Delta}$)	TMX	S4	Not evaluated	Not evaluated	No change in body or muscle mass
mdx:Cdh5^{CreERT2}*:Flt1*$^{LoxP/LoxP}$ (*mdx:Cdh5-Flt1*$^{\Delta/\Delta}$)	TMX	3, 4, S5	Increased capillary density	Improved muscle pathology	Improved grip strength
mdx	Anti-FLT1 peptide	5, S6, S7	Increased capillary density	Improved muscle pathology	Improved grip strength
mdx	PEG-Anti-FLT1 peptide	S8	No change	No change	No change
mdx	Anti-FLT1 MAb (MAB0702)	6, 7, S9	Increased capillary density	Improved muscle pathology	Improved grip strength
mdx	Anti-FLT1 MAb (EWC)	6, 7, S9	No change	No change	No change in body weight
