# Supplementary material for: Inhibition of FLT1 ameliorates muscular dystrophy phenotype by increased vasculature in a mouse model of Duchenne muscular dystrophy
Source: PLoS Genet. 2019 Dec 26;15(12):e1008468. doi: 10.1371/journal.pgen.1008468 (PMC6932757; doi:10.1371/journal.pgen.1008468)
Supplement: S2 Table — (PDF) [file pgen.1008468.s011.pdf]

Table S2

Table S2. Biacore analysis for affinity of monoclonal antibodies against FLT1			
**Mouse FLT1**	**Ka ($M^{-1}s^{-1}$)**	**Kd (s^{-1})**	**KD (M)**
MAB0702	5.555E+05	1.696E-03	3.053E-09
EWC	4.674E+05	3.488E-06	7.463E-12
**Human FLT1**	**Ka ($M^{-1}s^{-1}$)**	**Kd (s^{-1})**	**KD (M)**
MAB0702	3.811E+05	3.234E-04	8.486E-10
EWC	1.036E+05	6.625E-04	6.397E-09
