# Supplementary material for: Inhibition of FLT1 ameliorates muscular dystrophy phenotype by increased vasculature in a mouse model of Duchenne muscular dystrophy
Source: PLoS Genet. 2019 Dec 26;15(12):e1008468. doi: 10.1371/journal.pgen.1008468 (PMC6932757; doi:10.1371/journal.pgen.1008468)
Supplement: S3 Table — (PDF) [file pgen.1008468.s012.pdf]

Table S3

**Table S3 DNA primer sequences**					
**Gene**	**Forward**	**Sequence**	**Reverse**	**Sequence**	**Product (bp)**
**Genotyping**					
Flt1$^{LoxP/LoxP}$	Common	GTGCCACTGACCTAACATGTAAGAG	Common	GCGAAAAACAGTCAGTAGAAGT	245 (WT) 280 (KI)
CAG^{CreERTM}	OIMR1084	GCGGTCTGGCAGTAAAAACTATC	OIMR1085	GTGAAACAGCATTGCTGTCACTT	102 (TG)
Cdh5^{CreERT2}	CRE-1	AACCTGGATAGTGAAACAGGGGC	ER-1	CTCCATGGAGCGCCAGACGAGACC	408 (TG)
**qPCR**					
Flt1	Exon 1	CTTGCTCACCATGGTCAGCTGCTG	Exon 2	CACTTTTAACTTCGACCCTGAGCC	103 (WT/KO)
Flt1	Exon 2	GGCCAGACTCTCTTTCTCAAGTGC	Exon 3	GCAGAATTGCCTGTTATCCCTCCC	135 (WT)
18S rRNA	18S-F1	CGCACGGCCGGTACAGTGAAACTG	18S-R1	CACCCGTGGTCACCATGGTAGGCA	343
